# Mental health resilience in the adolescent offspring of parents with depression: a prospective longitudinal study

**DOI:** 10.1016/S2215-0366(15)00358-2

**Published:** 2016-01

**Authors:** Stephan Collishaw, Gemma Hammerton, Liam Mahedy, Ruth Sellers, Michael J Owen, Nicholas Craddock, Ajay K Thapar, Gordon T Harold, Frances Rice, Anita Thapar

**Affiliations:** a Child and Adolescent Psychiatry Section, Institute of Psychological Medicine and Clinical Neurosciences, Cardiff University School of Medicine, Cardiff, UK; b MRC Centre for Neuropsychiatric Genetics and Genomics, Cardiff University School of Medicine, Cardiff, UK; c Rudd Centre for Adoption Research and Practice, School of Psychology, University of Sussex, Brighton, UK; d International Center for Research in Human Development, Tomsk State University, Tomsk, Russia; e MRC Social, Genetic and Developmental Psychiatry Centre, Institute of Psychiatry, Psychology and Neuroscience, Kings College London, London, UK

## Abstract

**Background:**

Young people whose parents have depression have a greatly increased risk of developing a psychiatric disorder, but poor outcomes are not inevitable. Identification of the contributors to mental health resilience in young people at high familial risk is an internationally recognised priority. Our objectives were to identify protective factors that predict sustained good mental health in adolescents with a parent with depression and to test whether these contribute beyond what is explained by parent illness severity.

**Methods:**

The Early Prediction of Adolescent Depression study (EPAD) is a prospective longitudinal study of offspring of parents with recurrent depression. Parents with recurrent major depressive disorder, co-parents, and offspring (aged 9–17 years at baseline) were assessed three times over 4 years in a community setting. Offspring outcomes were operationalised as absence of mental health disorder, subthreshold symptoms, or suicidality on all three study occasions (sustained good mental health); and better than expected mental health (mood and behavioural symptoms at follow-up lower than predicted given severity of parental depression). Family, social, cognitive, and health behaviour predictor variables were assessed using interview and questionnaire measures.

**Findings:**

Between February and June, 2007, we screened 337 families at baseline, of which 331 were eligible. Of these, 262 completed the three assessments and were included in the data for sustained mental health. Adolescent mental health problems were common, but 53 (20%) of the 262 adolescents showed sustained good mental health. Index parent positive expressed emotion (odds ratio 1·91 [95% CI 1·31–2·79]; p=0·001), co-parent support (1·90 [1·38–2·62]; p<0·0001), good-quality social relationships (2·07 [1·35–3·18]; p=0·001), self-efficacy (1·49 [1·05–2·11]; p=0·03), and frequent exercise (2·96 [1·26–6·92]; p=0·01) were associated with sustained good mental health. Analyses accounting for parent depression severity were consistent, but frequent exercise only predicted better than expected mood-related mental health (β=–0·22; p=0·0004) not behavioural mental health, whereas index parents' expression of positive emotions predicted better than expected behavioural mental health (β=–0·16; p=0·01) not mood-related mental health. Multiple protective factors were required for offspring to be free of mental health problems (zero or one protective factor, 4% sustained good mental health; two protective factors, 10%; three protective factors, 13%, four protective factors, 38%; five protective factors, 48%).

**Interpretation:**

Adolescent mental health problems are common, but not inevitable, even when parental depression is severe and recurrent. These findings suggest that prevention programmes will need to enhance multiple protective factors across different domains of functioning.

**Funding:**

Sir Jules Thorn Charitable Trust, Economic and Social Research Council.

## Introduction

Depression is common, familial, often recurrent, and one of the world's leading causes of disability burden.^1^ Offspring of parents with depression are at three-to-four times higher risk of developing a wide range of mental health disorders than are offspring of non-depressed parents, with adverse health, educational and social outcomes, including increased risk of suicide.^1^, ^2^ Mental health disorders are a global problem in children and adolescents,^3^ in whom they show persistence into adulthood and have lifelong consequences.^4^ Effective prevention of mental health disorders in this identifiable high-risk group is therefore important.^5^

One approach to improving outcomes is to ameliorate the risk to which young people are exposed. Trials of treatment of adult depression show potential benefits for offspring mental health,^6^ although not all parents respond to treatment and, even when they do, positive mental health effects on children are not always seen.^7^ An alternative approach is to provide preventive interventions for at-risk adolescent offspring themselves.^8^

Typically, prevention strategies are informed by observational research on risks and adverse outcomes.^9^ However, many at-risk offspring show remarkably positive mental health outcomes without intervention.^10^, ^11^ Understanding what explains young people's resilience in the context of familial risk is important for identifying additional prevention targets.^12^

**Research in context**

**Evidence before this study**

Previous research has typically examined risk mechanisms that explain increased psychopathology in children of parents with depression as compared to non-depressed parents. We undertook a systematic search on Dec 5, 2014, using the Web of Science database and the search terms “resilience” and (“maternal depression” or “paternal depression”, or “parental depression”) and (“child” or “adolescent”). We identified additional papers by checking citations and cited papers. We found only three studies on adolescent offspring of parents with depression that used longitudinal designs to test predictors of mental health disorder absence. These studies differed from the present study in that variation in severity of parental mental illness risk exposure was not taken into account, nor was type of offspring outcome examined.

**Added value of this study**

The present study focused on factors that account for resilience in high-risk adolescents, and to our knowledge is the first to show that child, family, social, and lifestyle factors together contribute to adolescent mental health resilience. Crucially, these protective effects are not merely markers of parental depression severity—a caveat that has not been accounted for in previous studies. The study findings are also novel in that they reveal different contributors to mood and behavioural resilience. Two important findings are that emotional support from the healthy co-parent and youth physical exercise contribute to adolescent mood-related resilience even when parental depression severity is taken into account.

**Implications of all the available evidence**

Adolescent mental health problems are common among offspring of parents who have recurrent depression, but our findings highlight that adolescent mental health problems in those at familial risk are not inevitable, and that interventions aimed at enhancing resilience will need to target and change multiple social and lifestyle factors. Evidence supports multimodal interventions for at-risk adolescents. Extension of family-focused aspects of interventions to include both parents may be of particular benefit. Providing information and support that encourages healthy lifestyles (including frequent exercise) and that encourages young people to capitalise on friendship networks also seem likely to be beneficial for maintaining good mental health.

Mental health resilience has been conceptualised in different ways.^13^ Most studies have compared subgroups of at-risk individuals who either do or do not have mental health problems.^11^, ^14^, ^15^ An alternative approach operationalises resilience as showing lower symptom scores than those predicted by measures of risk. This approach has several important advantages as discussed by others.^13^, ^16^, ^17^ First, resilience is defined in terms of better than expected—rather than simply good—adaptation. This ensures that identified protective factors are not simply markers of lower severity of risk. Second, it permits distinction of protective factors for different mental health outcomes (eg, mood as well as behavioural). To our knowledge, no studies have used this approach to investigate resilience in offspring of parents with depression to date.

Family, social, and cognitive factors suggested to be associated with mental health resilience in young people include good-quality relationships with the parent with depression, support provided by other family members and friends, and adolescents' own self-appraisal.^10^, ^11^, ^12^, ^13^, ^14^, ^15^ Some national guidelines (eg, such as those of the National Institute for Health and Care Excellence^19^) also highlight potential protective effects of frequent physical exercise for depression.^18^, ^19^ However, we do not know whether these factors simply reflect lower levels of familial risk exposure. Also, although promoting mental health resilience in young people at familial risk is an internationally recognised priority,^5^ is it enough for prevention programmes to focus on a single domain of functioning?

This study examines adolescent offspring of parents with recurrent depression, studied prospectively in adolescence. We first examined the subgroup of at-risk individuals who exhibited no mental health problems for the duration of the study; then we used a residual scores method to assess family, social, and cognitive predictors of better-than-expected mood and behaviour outcomes beyond that accounted for by severity of parental depression.

## Methods

### Study design and participants

The Early Prediction of Adolescent Depression study (EPAD) is a prospective longitudinal study of offspring of parents with recurrent depression.^20^ Families were recruited primarily from general practices across South Wales, UK. The presence of at least two previous episodes of parent DSM-IV major depressive disorder was confirmed at baseline interview. The youngest child in the age range 9–17 years was selected for inclusion. All selected children were biologically related to and living with the affected parent. We excluded offspring with an intelligence quotient lower than 50, children with serious physical illnesses, and parents with psychosis, bipolar disorder, or mania or hypomania. Parents and young people provided written informed consent (≥16 years of age) or assent (<16 years). The Multicentre Research Ethics Committee for Wales provided ethical approval.

### Procedures

Assessments were undertaken on three occasions (referred to as waves), 12–18 months apart, over a 4-year period (2007–11). Trained, supervised research psychologists assessed families at home using semi-structured research diagnostic interviews and self-report questionnaires to measure risk exposure, mental health of adolescents, and family, social, cognitive, and health behaviour protective factors.

For risk exposure, we looked at the severity and course of parental depression. Parents were interviewed at each of the three waves with the Schedules for Clinical Assessment in Neuropsychiatry (SCAN)^21^ to assess past month episodes of depression and to collect information about additional episodes between assessments. Interviews at baseline ascertained parents' age at first episode, periods of hospital admission for depression, impairment of the worst two episodes using Global Assessment of Functioning scores,^22^ and additional family history of depression (in adolescents' siblings, parents, and grandparents). We also retrospectively obtained information about depression during pregnancy and the postnatal period (up to 1 year after birth) with the index child from the mothers in the sample.

To assess the mental health of the adolescent, we used the Child and Adolescent Psychiatric Assessment (CAPA) interview and a questionnaire. The CAPA is a well validated semi-structured diagnostic interview.^23^ It assesses psychiatric symptoms and disorders over the preceding 3 months according to DSM-IV criteria.^22^ Sections on “mood” (depressive disorders), “behaviour” (oppositional defiant disorder and conduct disorder), anxiety disorders, attention-deficit hyperactivity disorder, bipolar disorder, cyclothymia, and eating disorders were completed independently with parents and young people at each wave. Two child and adolescent psychiatrists reviewed the diagnosed and sub-threshold cases. CAPA interviews also generated mood and behaviour disorder symptom totals. Inter-rater reliabilities for symptoms were excellent (average κ=0·94). Suicidality or self-harm was coded if parents or adolescents reported suicidal ideation or behaviour in the CAPA or endorsed the item “thought about killing self” on a well validated child and adolescent depression measure, the Mood and Feelings Questionnaire.^24^

Parents and adolescents also completed the well validated Strengths and Difficulties Questionnaire (SDQ),^25^ a 25-item screen for common emotional and behavioural problems allowing a direct comparison with UK population norms.^26^

The family, social, cognitive, and health behaviour protective factors were assessed at baseline unless otherwise specified.

For family functioning, we looked at four measurements. First, we assessed index parent-rated warmth towards the adolescent using the Iowa Youth and Families Project (IYFP) parental warmth subscale (six items, range 6–42, α=0·93).^27^ Second, we recorded “five minute expressed emotion” interviews, and trained researchers coded positive expressed emotions of index parents about adolescents according to tone and content of speech samples (range 0–5), as previously validated.^28^ We imputed partial missing data for this particular task at baseline using expressed emotion data at first follow-up. Third, adolescents rated co-parent emotional support using the interviewer-administered Perceived Social Support scale (eg, “this person listens if I need to talk about worries”; range 0–6; α=0·95).^29^ Finally, we measured sibling warmth using the IYFP family interaction rating scales (six items, range 6–30, α=0·84).^27^

For social relationships and friendships, we looked at four measurements. First, we used the parent-rated five-item SDQ peer subscale, which is a measure commonly used in epidemiological surveys to assess positive and negative aspects of young people's social relationships (eg, “liked by other children”, “has at least one good friend”).^25^ Negative items were reverse coded. Higher total scores indicated better quality social relationships (range 0–10; α=0·68). Second, we used the adolescent-rated SDQ peer subscale coded in the same way (range 0–10, α=0·55). Third, we assessed adolescent-perceived friendship quality using a ten-item questionnaire assessing social esteem and peer inclusion (eg, “other children think I am a nice person”, “other children want to be my friend”; α=0·83).^30^ Finally, parents also reported attendance at clubs or other organised out-of-school activities (at least monthly).

For adolescent exercise, we assessed the frequency of exercise using an adolescent questionnaire at baseline with two items: “how often do you exercise (intense enough to be out of breath)?” and “how often do you play sport?”. Ratings were combined into a single dichotomous indicator (intense exercise or sport more than once a week *vs* less often).^19^ We first assessed adolescent-reported self-efficacy at wave 2 using the ten-item Generalized Self Efficacy Scale (α=0·98). This measure assesses young people's perceived ability to overcome problems, cope with adversity and achieve difficult tasks (eg, “if I am in trouble, I can usually think of a solution”).^31^

### Statistical analysis

Logistic regression analyses examined associations between protective factors and sustained good mental health in offspring. This outcome variable was defined as the absence at all three waves of any DSM-IV disorder diagnosis, of elevated CAPA interview depression or behaviour disorder symptoms (both three or more), or of suicidal ideation or self-harm. Interactions with gender and age were also tested. Cumulative effects models tested the extent to which significant predictors jointly contributed to sustained good mental health. For this purpose, we dichotomised protective factors using standard cutpoints or otherwise median splits.

We repeated the analyses for subgroups of families in which parent severity differed: depressive episode recurrence over the study, yes or no; past severe depressive episode (Global Assessment of Functioning score <30 or admitted to hospital), yes or no.

We created the continuous outcomes of mood resilience and behavioural resilience using residual scores generated via regression analysis. Adolescent mood disorder and behaviour disorder symptom counts at final follow-up were regressed onto the predictor variables indexing parent illness-related risks (parent depression age at onset, parent depression severity, family history of depression). Negative residual scores indicate better than predicted offspring mood and behaviour (resilience) at follow-up and allow for variability in the level of parental depression-related risk.^16^, ^17^ Univariate and multivariate models tested associations between hypothesised protective factors and the two derived continuous outcome measures of mood and behavioural resilience.

### Role of the funding source

The funders had no role in the study design, data collection, data analysis, data interpretation, or writing of the report. The corresponding author had full access to all the data in the study and had final responsibility for the decision to submit for publication.

## Results

**Figure 1:**
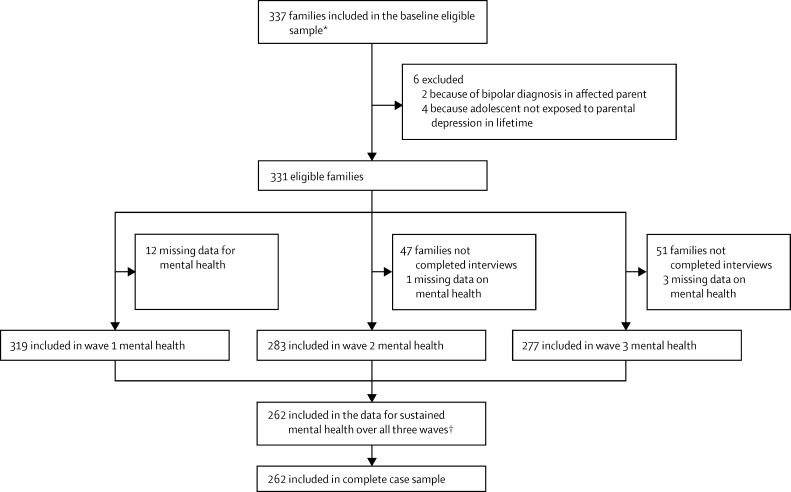
Retention of participants in the Early Prediction of Adolescent Depression study (EPAD) Initial telephone screening identified 469 families. 116 families withdrew before baseline assessment, or were withdrawn for other reasons (incomplete baseline assessments, bipolar diagnosis at baseline assessment; n=16). The baseline eligible sample thus consisted of 337 participants. *Participants were recruited primarily from 62 general practitioner surgeries (263 of 337 of eligible baseline sample), from a database of previously identified adults with recurrent unipolar depression from the community (64 of 337), and via other methods (posters in local health centres, and depression alliance newsletter; 10 of 337). †Numbers vary in main analyses from 209 to 260 due to missing data on individual protective factors.

Of the 469 families screened between February and June, 2007, we included 337 index parents with recurrent major depressive disorder (315 women and 22 men) and adolescent offspring who were biologically related to and living with the affected parent (figure 1). Six families were excluded at follow-up because of bipolar disorder in the parent with depression (n=2) or because adolescents had not been exposed to episodes of parental depression in their lifetime (n=4), so the final number of families included in the eligible sample was 331 (194 girls and 137 boys, mean age 12·4 years [SD 2·0] at baseline). Table 1 shows the demographic characteristics of the eligible sample at baseline.

Full information on offspring mental health was available for 262 (79%) of the 331 eligible baseline sample. We present the results for the analysis using the complete case sample (n ≤262). Sensitivity analyses used multiple imputation (see the appendix for information on characteristics of sample retained *vs* not retained, details of the imputation, and imputed results). Results for imputed data were closely similar.

Overall, 103 (39%) of 262 adolescents met criteria for a psychiatric diagnosis, 118 (45%) had elevated depression symptoms, 182 (70%) elevated behaviour symptoms, and 73 (28%) exhibited suicidal ideation or self-harm on at least one occasion. 53 (20%) exhibited none of these mental health problems across the study period and thus met study criteria for sustained good mental health (table 2).

The likelihood of sustained good mental health did not differ by adolescent gender or age, but was lower for adolescents whose parents had a history of severe depressive episodes (table 3). SDQ screen scores indicated equivalent or better mental health for those with no mental health problems than for the UK population for this age group (appendix).

Family, social, and adolescent cognitive or health behaviour factors were associated with sustained good mental health in offspring (table 4). Positive expressed emotion in index parents, co-parent support, parent-rated peer relationship quality, adolescent self-efficacy, and frequent exercise were all associated with good mental health. Index-parent warmth, sibling warmth, out-of-school activities, and adolescent-rated peer relationship and friendship quality were not significant predictors. These bivariate associations did not differ according to gender or age (all interactions non-significant, p>0·05).

**Figure 2:**
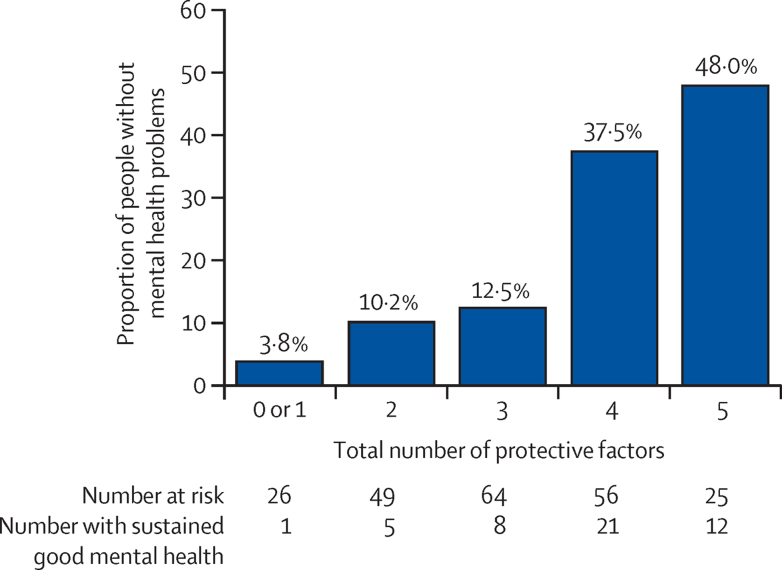
Cumulative influences on sustained good mental health (n=220) Likelihood of sustained good mental health in offspring according to total number of identified protective factors: positive index parent expressed emotion (high or very high), coparent support (median split; score >3), good-quality social relationships (parent Strengths and Difficulties Questionnaire peer subscale in normal range), adolescent efficacy (median split; General Self Efficacy Scale >28), physical exercise (intense exercise or sport more often than once per week)

The likelihood of sustained good mental health in the offspring increased with the total number of significant protective factors present across family, social, and adolescent cognitive or health behaviour domains (odds ratio, OR=2·27 [1·62–3·19], p<0·0001; figure 2). The proportion of adolescents with sustained good mental health ranged from 3·8% for zero or one protective factor to 48·0% for five protective factors.

As a sensitivity check, analyses investigated whether protective effects varied between families where parents had (n=167) or had not (n=93) experienced an episode recurrence by follow-up, or had (n=73) or had not (n=186) experienced a past severe episode of depression (Global Assessment of Functioning score <30 or hospital admission). The number of protective factors was associated with offspring mental health irrespective of parent depression episode recurrence (association within subgroups: recurrence, OR=2·45 [1·56–3·83], p<0·0001; no recurrence, OR=1·98 [1·17–3·37], p=0·011; interaction: OR=1·23 [0·62–2·47], p=0·56). The association between number of protective factors and offspring sustained mental health was significant for those not exposed to a severe parent depression episode (OR=2·23 [1·53–3·24], p<0·0001) but not in the subgroup exposed to a severe episode (OR=2·67 [0·91–7·87], p=0·075), although the ORs did not differ significantly between the two subgroups (interaction OR=1·20 (0·38–3·76), p=0·76).

Table 5 shows univariate tests of association with mood and behavioural resilience. Negative residuals suggested lower than expected mood and behaviour symptom scores given parental depression severity. Baseline co-parent support (but not index parent factors), better parent-rated and adolescent-rated social relationships, and adolescent self-efficacy were associated both with mood and behavioural resilience at the final assessment. Frequent exercise was associated with mood resilience only, whereas index parent warmth and positive expressed emotion were associated with behavioural resilience only. Two multivariate models of mood and behaviour resilience were then examined taking forward significant univariate protective factors (appendix). The first model found that co-parent support (β=–0·19, p=0·004), adolescent self-efficacy (β=–0·19, p=0·004), and adolescent exercise (β=–0·17, p=0·01) independently predicted mood resilience, with a marginal effect of parent-rated peer relationship quality (β=–0·14, p=0·05). The second showed that parent-rated peer relationship quality (β=–0·16, p=0·04) and adolescent self-efficacy (β=–0·21, p=0·004) independently predicted behavioural resilience.

When we did our sensitivity analyses, we saw that all results were comparable when excluding male index parents (n=19) from the sample with sustained mental health information (data not shown). When additionally excluding offspring not living with their father at baseline (n=73) findings were similar for associations between co-parent (ie, paternal) support with offspring sustained mental health (OR=1·93 [1·31–2.81], p=0·001) and mood resilience at follow-up (β=–0·22, p=0·006). However, there was no longer evidence of an association between paternal support and behavioural resilience (β=–0·15, p=0·07). The multivariate model results for mood and behavioural resilience remained identical using alternative forward and backward step-wise regression that included all predictor variables. Finally, analyses were repeated using multiple imputation to address missing data. Results were closely comparable (appendix).

## Discussion

Our findings show that, as a group, offspring of parents with recurrent depression experienced high rates of mental health problems. Despite this finding, about one in five adolescents had sustained good mental health across all three waves of assessment. Index-parent positive expressed emotion, support from co-parents, good quality social relationships, youth self-efficacy, and regular physical exercise all predicted sustained good mental health. Protective factors also predicted lower than expected youth mood and behaviour symptoms at follow-up given the severity of their parents' depression.

The findings of this study are important because they highlight several novel protective factors associated with resilience in adolescents at high risk of psychiatric problems due to recurrent parental depression. The findings advance our understanding by directly taking account of variation in the severity of parents' depression and by considering protective effects across multiple domains including co-parent support and adolescent physical exercise that have not been previously investigated. The results are also novel in highlighting that multiple protective factors are required, and in showing differential protection for mood and behavioural resilience.

Depressive disorder in adults who are parents is common, as is cross-generational transmission of mental health problems. However, our findings have shown that mental health problems in this high-risk group of young people are not inevitable, even when parents have experienced multiple episodes of major depressive disorder. Against a background of significantly elevated risk for psychopathology, including depression, behaviour problems, and suicidality, a subgroup of offspring (20%) was characterised by a pattern of sustained good mental health in adolescence and remained problem free for the duration of this study. This subgroup showed better or equivalent levels of mental health when compared with age-appropriate general population norms for the SDQ.

The identification of factors associated with good mental health in adolescents who are at high familial risk has important implications for treatment and prevention. Findings from this study show that family, social, cognitive, and health behaviour factors contributed to resilience, when defined as sustained good adolescent mental health, but that multiple protective factors were required. Even in this clinically derived sample of parents with recurrent depression there was substantial variation in parent illness course and severity. Crucially, we were able to also show that protective effects did not simply reflect variation in parental depression severity. Specifically we accounted for parent age at onset and severity of depression as well as family history of depression to assess better than expected functioning given the severity of risk exposure. Our results showed that co-parent support, social factors, and adolescents' self-efficacy each predicted both mood and behavioural resilience. The study findings are also novel in that they reveal that contributors to mood and behavioural resilience differ. Interestingly, frequent physical exercise and co-parent emotional support were found to have a specific association with mood resilience only.

Co-parent emotional support emerged as a particularly strong predictor of mental health resilience in this high-risk sample. Most of the index parents in this sample were mothers, and the present findings show the importance of father involvement in moderating the potential impact of maternal depression. The reasons why most of our sample consisted of mothers with depression may include gender differences in prevalence of adult depression^32^ and in help-seeking and presentation in primary care, as well as a greater likelihood for mothers with depression to opt in to our study once approached. These findings highlight the potential benefits of including the wider family in prevention programmes for adolescent depression.^12^

Research has shown protective effects of good-quality social relationships in relation to psychosocial adversities such as maltreatment.^33^ We showed that there were also strong associations with mental health resilience in offspring of parents with depression.

Extending previous findings,^14^, ^15^ self-efficacy was not only a strong predictor of sustained mental health, but also of mood and behaviour resilience at follow-up. Belief in one's ability to successfully deal with adversity might be especially important in the context of parental depression if it allows young people to better rationalise their parent's illness, and exert greater control over their own responses to stressors that result from parent illness.^13^, ^15^

Finally, frequent physical exercise was associated with lower than predicted depression problems. This supports National Institute for Health and Care Excellence guidance that regular intense exercise is advised to ameliorate or prevent depression, evidence that has thus far been lacking for young people.^19^

The findings suggest that there are potential modifiable targets for preventative interventions that might help interrupt the intergenerational transmission of risk for psychopathology between parents with depression and their children. Treatment of parents is a priority but might be insufficient on its own to prevent psychopathology in offspring. Treatment studies have shown that remission of parental depression is associated with reductions in some types of offspring symptoms. However, not all parents with depression seek treatment, and treatment is not always successful. Furthermore, likelihood of success is associated with parental depression severity (controlled for in this study).^7^, ^8^

The study highlights several additional targets for preventative intervention beyond risk reduction. These include the facilitation of support from co-parents and young people's social relationships. Cognitive behavioural programmes that enhance adolescents' sense of efficacy together with programmes to promote healthy lifestyles, including exercise, are also likely to be important.

A number of cognitive-behaviourally orientated prevention programmes already exist. These seem to be effective for some children and adolescents in high-income and low-income countries, but importantly previous evidence also suggests such prevention programmes are ineffective when a parent is currently depressed.^8^ Careful thought is required in deciding about which programmes to target at which young people.^34^ One important message from the present study is that efforts to target single protective factors in isolation for at-risk adolescents are probably insufficient. Good mental health outcomes were typically achieved only if adolescents reported a combination of multiple protective factors across family, peer, and cognitive or behavioural domains. The results support the idea that multimodal interventions that simultaneously address multiple systems (eg, home, school, and the young person themselves) hold the greatest promise for preventing mental ill health.^35^, ^36^

Our findings are also relevant to adult mental health services. Raising awareness of the importance of early prevention of mental health problems in the offspring of parents with depression is important, as is enhancing effective links between adult and youth mental health services.

The study used a large longitudinal sample of parents with depression and adolescents, well validated multi-informant assessments of psychiatric disorders and subthreshold problems, together with careful characterisation of parental depression risk and hypothesised resilience promoting factors.

This study has also limitations. First, further follow-up will be needed to determine whether resilience is sustained into early adulthood. Second, even carefully designed longitudinal observational studies cannot unambiguously identify causal influences, because of the possible role of other unmeasured confounders or reverse causation. Finally, we cannot rule out the contribution of genetic factors that were not indexed by parent illness severity.

In conclusion, depression is familial, and, although psychiatric problems among adolescents whose parents have recurrent depression are common, they are not inevitable. Some young people show unexpectedly positive outcomes. The study identified several potentially modifiable protective factors that together seem to promote adolescents' mental health resilience. These findings now need to be taken forward by refining existing preventive interventions, developing new ones, and testing through randomised trials.

## Figures and Tables

**Table 1:** Parent and child demographic characteristics of the eligible sample at baseline

	**Number of individuals (%) or mean (SD), n=331**
**Parent characteristics**	
Female	309 (93%)
Age at baseline, years	41·6 (5·4)
Single parent	95 (29%)
Family income below £20 000	91 (30%)
**Child characteristics**	
Female	194 (59%)
Age at baseline, years	12·4 (2·0)
IQ	94·9 (12·9)

IQ=intelligence quotient.

**Table 2:** Offspring without mental health problems at each wave and by gender

	**Number of participants (%)**
**Boys**	
Baseline assessment (n=131)	48 (37%)
First follow-up (n=111)	43 (39%)
Second follow-up (n=115)	45 (39%)
Across the study period (n=105)	21 (20%)
**Girls**	
Baseline assessment (n=188)	61 (32%)
First follow-up (n=172)	70 (41%)
Second follow-up (n=162)	59 (36%)
Across the study period (n=157)	32 (20%)

**Table 3:** Offspring of parents with depression: mental health according to differences in risk exposure and adolescent characteristics

	**Sustained good mental health (n=53)**	**No sustained good mental health (n=209)**	OR (95% CI)*	**p value**
**Index parent depression severity**				
Age at first episode, years	29·16 (9·33)	25·68 (8·21)	1·49 (1·10–2·02)	0·010
Worst episode hospitalisation or GAF <30	5/52 (10%)	68/207 (33%)	0·22 (0·08–0·57)	0·002
Recurrence during study period	31/53 (58%)	136/207 (66%)	0·74 (0·40–1·36)	0·33
Antenatal depression	3/46 (7%)	22/194 (11%)	0·55 (0·16–1·91)	0·34
Postnatal depression	18/46 (39 %)	84/194 (43%)	0·84 (0·44–1·62)	0·61
**Additional family history of depression**				
Two or more first-degree or second-degree relatives	20/53 (38%)	83/209 (40%)	0·92 (0·50–1·71)	0·79
**Adolescent characteristics**				
Age, years	12·32 (2·09)	12·39 (2·03)	0·97 (0·72–1·31)	0·83
Female	32/53 (60%)	125/209 (60%)	1·02 (0·55–1·90)	0·94

Data are n (%) or mean (SD). Total numbers vary because of occasional missing data for measures of index parent severity measures. OR=odds ratio. GAF=global assessment of functioning.

* For analyses of parent age at onset and offspring age, ORs indicate change in odds of sustained good mental health per one SD change in mean age.

**Table 4:** Univariate associations of family, social and adolescent cognitive/health behaviour factors with sustained good adolescent mental health

	**Sustained good mental health (n=53)**		**No sustained good mental health (n=209)**		OR (95% CI)*	**p value**
	N	n (%) or mean (SD)	N	n (%) or mean (SD)		
**Family factors**						
Index parent warmth	53	36·74 (5·75)	200	35·84 (6·08)	1·19 (0·84–1·69)	0·34
Index parent positive expressed emotion	52	3·85 (0·75)	206	3·33 (1·00)	1·91 (1·31–2·79)	0·0008
Co-parent support to adolescent	52	3·79 (2·73)	208	2·08 (2·61)	1·90 (1·38–2·62)	<0·0001
Sibling warmth	40	17·00 (4·86)	169	16·38 (4·92)	1·14 (0·80–1·61)	0·48
**Social factors**						
Parent-reported peer relationship quality	53	9·00 (1·43)	202	7·91 (2·12)	2·07 (1·35–3·18)	0·001
Adolescent-reported peer relationship quality	51	8·45 (1·47)	201	7·97 (1·82)	1·36 (0·96–1·93)	0·08
Out of school activities (monthly)	50	33 (66%)	197	114 (58%)	1·41 (0·74–2·71)	0·30
Adolescent perceived friendships	52	28·42 (5·02)	197	27·04 (5·81)	1·30 (0·94–1·81)	0·12
**Adolescent self-efficacy and exercise**						
Self efficacy	48	29·19 (3·02)	186	27·46 (5·06)	1·49 (1·05–2·11)	0·03
Frequent physical exercise	52	45 (87%)	200	137 (69%)	2·96 (1·26–6·92)	0·01

Data are mean (SD) or n (%), unless otherwise specified. For scale scores, ORs indicate change in odds per one SD change in mean scale score.

* Ns for predictor variables range from 209 to 260. Data from multiple imputation models shown in supplementary table 1.

**Table 5:** Univariate associations of family, social and adolescent cognitive or health behaviour factors with mood and behaviour resilience at final follow-up

	**Standardised residuals Mood resilience**			**Standardised residuals Behavioural resilience**		
	N	β	p value	N	β	p value
**Family factors**						
Index-parent warmth	260	−0·06	0·33	256	−0·17	0·007
Index-parent positive expressed emotion	261	−0·11	0·08	257	−0·16	0·01
Co-parent support	268	−0·23	0·0001	264	−0·14	0·03
Sibling warmth	211	0·06	0·43	208	−0·10	0·15
**Social Factors**						
Parent-reported peer relationship quality	260	−0·17	0·006	256	−0·23	0·0002
Adolescent-reported peer relationship quality	256	−0·17	0·005	253	−0·16	0·01
Out of school activities	251	−0·15	0·02	248	−0·10	0·12
Adolescent perceived friendships	253	−0·13	0·03	250	−0·15	0·02
**Adolescent cognition or behaviour**						
Self-efficacy	228	−0·22	0·001	224	−0·25	0·0001
Frequent physical exercise	256	−0·22	0·0004	253	−0·001	0·99

*N*s for predictor variables and outcome data range from 208 to 268. Data from multiple imputation models shown in supplementary table 2.
