# Supplementary material for: Mental health resilience in the adolescent offspring of parents with depression: a prospective longitudinal study
Source: Lancet Psychiatry. 2016 Jan;3(1):49–57. doi: 10.1016/S2215-0366(15)00358-2 (PMC4703896; doi:10.1016/S2215-0366(15)00358-2)
Supplement: Supplementary appendix [file mmc1.pdf]

THE LANCET Psychiatry

## Supplementary appendix

This appendix formed part of the original submission and has been peer reviewed. We post it as supplied by the authors.

Supplement to: Collishaw S, Hammerton G, Mahedy L, et al. Mental health resilience in the adolescent offspring of parents with depression: a prospective longitudinal study. *Lancet Psychiatry* 2015; published online Dec 1. http://dx.doi.org/10.1016/S2215-0366(15)00358-2.

*Supplementary Figure.* A comparison of mental health with population norms.

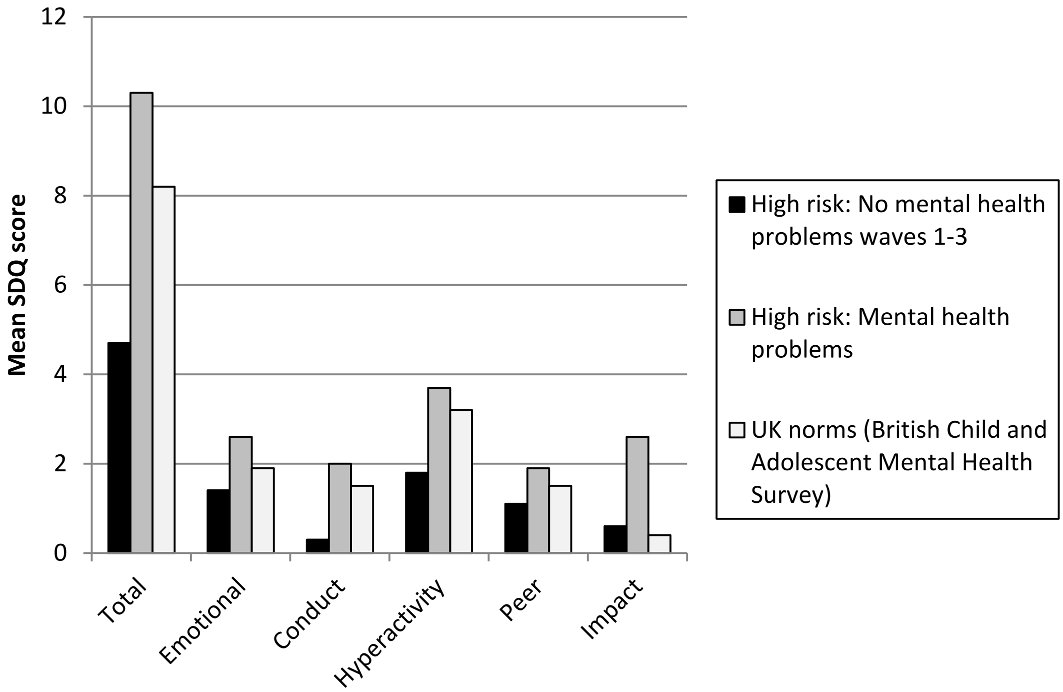


Parent-rated Strength and Difficulties Questionnaire (SDQ) screen scores at wave 3 (final follow-up) were compared for high risk adolescents with mental health problems (*n*=201), without mental health problems ('sustained good mental health', *n*=52), and a general population norm sample (British Child and Adolescent Mental Health Survey 1999, aged 11-15 years, *n*=4228).[26] SDQ total problem scores at wave 3 (see Figure) and at each of the other waves (not shown) were lower for those with sustained good mental health than for other adolescents in the high risk sample, and also lower than UK population norms. In addition, as shown in the Figure, this was true for all SDQ subscales.

*Missing data checks and sensitivity analyses using multiple imputation*

Families retained and lost to follow-up did not differ in terms of parent or adolescent age, adolescent gender, rates of adolescent DSM-IV disorder or suicidal ideation, but adolescents retained in the study had fewer baseline depression symptoms (1·6 vs 2·1; $p$=0·04), higher cognitive ability (IQ=96·8 vs. 87·3; $p$<0·001), and fewer parent-reported depression symptoms (2·4 vs. 3·6, $p$=0·001). Sensitivity analyses accounted for missing data for offspring mental health, protective factors and other covariates using multivariate imputation by chained equations.[S1] This assumes that data are missing at random (MAR) i.e. given the observed data included in the imputation model, the missingness mechanism does not depend on the unobserved data.[S2] The variables associated with non-response were therefore included in the imputation model to make the assumption of MAR as plausible as possible, along with multiple measures of offspring psychopathology and all other variables included in analyses.[S2] Imputation models were run using binary logistic and linear regression models as appropriate. Predictive Mean Matching (PMM) was used when continuous variables were not normally distributed. All variables with missing data used in analyses were imputed up to the maximum sample size of $n$=331 (apart from sibling warmth which was not imputed when offspring did not have a sibling). Fifty imputed datasets were derived each with 10 cycles of regression switching and then all analyses were run on imputed datasets by combining estimates using Rubin's rules. [S2] Results for the univariate associations are shown in supplementary tables S2 and S3, and are closely comparable to complete case analyses reported in the main text.

S1 Van Buuren, S & Oudshoom C. MICE: Multivariate imputation by chained equations (S software for missing data imputation). 2000.

S2 White IR, Royston P, Wood AM. Multiple imputation using chained equations: Issues and guidance for practice. Stat Med. 2011;30(4):377–99

*Supplementary Table S1*. Univariate associations with adolescent sustained good mental health using imputed data (*n*=331)

| | "Sustained good mental health" | |
|---|---|---|
| | OR (95% CI) [1] | *p* |
| Family Factors | | |
| Index parent warmth | 1·27 (0·91, 1·77) | 0·15 |
| Index parent positive expressed emotion | 1·79 (1·24, 2·59) | 0·002* |
| Co-parent support to adolescent | 2·01 (1·47, 2·75) | <0·001* |
| Sibling warmth[2] | 1·09 (0·78, 1·52) | 0·62 |
| Social Factors | | |
| Parent-reported peer relationship quality | 2·16 (1·41, 3·32) | <0·001* |
| Adolescent-reported peer relationship quality | 1·40 (1.00, 1.97) | 0·05 |
| Out of school activities (monthly) | 1·72 (0·92, 3·21) | 0·09 |
| Adolescent perceived friendships | 1·29 (0·95, 1·74) | 0·10 |
| Adolescent self efficacy and exercise | | |
| Self efficacy | 1·42 (1·02, 1·98) | 0·04* |
| Frequent physical exercise | 2·56 (1·14, 5·76) | 0·02* |

[1] For scale scores, ORs indicate change in odds per one SD change in mean scale score; [2] Sibling warmth run for only those adolescents who had a sibling (*n*=260)

*Supplementary Table S2.* Univariate associations with mood and behaviour resilience at final follow-up using imputed data (*n*=331)

| | Standardised residuals | | | |
|---|---|---|---|---|
| | Mood resilience | | Behavioural resilience | |
| | β | *p* | β | *p* |
| **Family Factors** | | | | |
| Index parent warmth | -0·05 | 0·39 | -0·18 | 0·004* |
| Index parent positive expressed emotion | -0·13 | 0·04* | -0·20 | 0·002* |
| Co-parent support | -0·22 | <0·001* | -0·14 | 0·02* |
| Sibling warmth[1] | 0·07 | 0·27 | -0·07 | 0·29 |
| **Social Factors** | | | | |
| Parent-reported peer relationship quality | -0·19 | 0·003* | -0·26 | <0·001* |
| Adolescent-reported peer relationship quality | -0·16 | 0·009* | -0·16 | 0·01* |
| Out of school activities | -0·19 | 0·001* | -0·10 | 0·11 |
| Adolescent perceived friendships | -0·12 | 0·06 | -0·11 | 0·08 |
| **Adolescent cognition/behaviour** | | | | |
| Self efficacy | -0·23 | <0·001* | -0·24 | <0·001* |
| Frequent physical exercise | -0·21 | <0·001* | -0·01 | 0·91 |

[1] Sibling warmth run for only those adolescents who had a sibling (*n*=260)

*Supplementary Table S3*. Multivariate models of mood and behaviour resilience at final follow-up[1]

| | Standardised residuals | | | | | |
|---|---|---|---|---|---|---|
| | Mood resilience (*n* = 208) | | | Behavioural resilience (*n* = 201) | | |
| | β | *SE* | *p* | β | *SE* | *p* |
| Family Factors | | | | | | |
| Index parent warmth | | | | -0·09 | 0·07 | 0·19 |
| Index parent positive expressed emotion | | | | -0·04 | 0·07 | 0·61 |
| Co-parent support | -0·19 | 0·06 | 0·004* | -0·09 | 0·06 | 0·20 |
| Social Factors | | | | | | |
| Parent-reported peer relationship quality | -0·14 | 0·08 | 0·05 | -0·16 | 0·08 | 0·04* |
| Out of school activities | -0·06 | 0·13 | 0·36 | | | |
| Adolescent perceived friendships | 0·07 | 0·07 | 0·34 | 0·01 | 0·08 | 0·86 |
| Adolescent cognition/behaviour | | | | | | |
| Self efficacy | -0·19 | 0·07 | 0·004* | -0·21 | 0·07 | 0·004* |
| Frequent physical exercise | -0·17 | 0·15 | 0·01* | | | |

[1]Models informed by significant univariate associations. *p<0·05. Mood resilience adjusted R-squared = 0·133; Behaviour resilience adjusted R-squared = 0·08; Multivariate model results using alternative backward and forward selection approaches showed equivalent results - Mood resilience (forward selection: self-efficacy, β=-.26, p<.001; co-parent support, β=-.19, p=.008; exercise: β=-.16, p=.02; backward selection: self-efficacy, β=-.27, p<.001; co-parent support, β=-.20, p=.004; exercise: β=-.16, p=.02); Behavioural resilience: (Forward selection: self efficacy: β=-.21, p=.004; parent rated peer: β = -.18, p=.01; Backward selection: self efficacy: β=-.21, p=.004; parent rated peer: β = -.18, p=.01).

*Supplementary Table S4.* Inter-correlations of predictor variables and mood and behaviour resilience at final follow-up

| | 1 | *2* | *3* | *4* | *5* | *6* | *7* | *8* | *9* | 10 | *11* | *12* |
|---|---|---|---|---|---|---|---|---|---|---|---|---|
| 1. Mood resilience | - | | | | | | | | | | | |
| 2. Behaviour resilience | .37** | - | | | | | | | | | | |
| 1. Index parent warmth | -.06 | -.17** | - | | | | | | | | | |
| 2. Ind parent positive expressed emotion | -.11 | -.16* | .33** | - | | | | | | | | |
| 3. Sib warmth | .06 | -.10 | .12 | .09 | - | | | | | | | |
| 6. Co-parent support | -.23** | -.14* | .06 | .03 | .10 | - | | | | | | |
| 7. Parent-reported peer relationship | -.17** | -.23** | .17** | .26** | .04 | -.01 | - | | | | | |
| 8. Adolescent-rated peer relationship | -.17** | -.16* | .06 | .10 | .09 | .01 | .57** | - | | | | |
| 9. Out of school activities[1] | -.15* | -.10 | .03 | .10 | -.05 | .13* | .17** | .08 | - | | | |
| 10. Adolescent perceived friendships | -.13* | -.15* | .03 | .07 | .12 | .04 | .36** | .52** | .15* | - | | |
| 11. Self efficacy | -.29** | -.24** | .12 | .19** | .11 | .14* | .10 | .15* | .17** | .23** | - | |
| 12. Frequent physical exercise[1] | -.22** | -.001 | .08 | .22** | .07 | .03 | .27** | .21** | .29** | .19** | .10 | - |

[1]Binary variables – point biserial and tetrachoric correlations estimated as appropriate; * p<0.05, **p<0.01
